# Risk Factors for SARS-CoV-2 Infection Among US Healthcare Personnel, May–December 2020

**DOI:** 10.3201/eid2801.211803

**Published:** 2022-01

**Authors:** Nora Chea, Cedric J. Brown, Taniece Eure, Rebecca Alkis Ramirez, Gregory Blazek, Austin R. Penna, Ruoran Li, Christopher A. Czaja, Helen Johnston, Devra Barter, Betsy Feighner Miller, Kathleen Angell, Kristen E. Marshall, Ashley Fell, Sara Lovett, Sarah Lim, Ruth Lynfield, Sarah Shrum Davis, Erin C. Phipps, Marla Sievers, Ghinwa Dumyati, Cathleen Concannon, Kathryn McCullough, Amy Woods, Sandhya Seshadri, Christopher Myers, Rebecca Pierce, Valerie L.S. Ocampo, Judith A. Guzman-Cottrill, Gabriela Escutia, Monika Samper, Nicola D. Thompson, Shelley S. Magill, Cheri T. Grigg

**Affiliations:** Centers for Disease Control and Prevention, Atlanta, Georgia, USA (N. Chea, C.J. Brown, T. Eure, R.A. Ramirez, G. Blazek, A.R. Penna, R. Li, K.E. Marshall, N.D. Thompson, S.S. Magill, C.T. Grigg);; Chenega Professional & Technical Services, LLC, Chesapeake, Virginia, USA (C.J. Brown, T. Eure);; Chenega Enterprise Systems & Solutions, LLC, Chesapeake (G. Blazek);; Colorado Department of Public Health and Environment, Denver, Colorado, USA (C.A. Czaja, H. Johnston, D. Barter, B.F. Miller, K. Angell, K.E. Marshall);; Minnesota Department of Health, St. Paul, Minnesota, USA (A. Fell, S. Lovett, S. Lim, R. Lynfield);; University of New Mexico, Albuquerque, New Mexico, USA (S.S. Davis, E.C. Phipps);; New Mexico Department of Health, Santa Fe, New Mexico, USA (M. Sievers);; University of Rochester Medical Center, Rochester, New York, USA (G. Dumyati, C. Concannon, K. McCullough, A. Woods, S. Seshadri, C. Myers);; Oregon Health Authority, Portland, Oregon, USA (R. Pierce, V.L.S. Ocampo, J.A. Guzman-Cottrill, G. Escutia, M. Samper)

**Keywords:** COVID-19, 2019 novel coronavirus disease, coronavirus disease, severe acute respiratory syndrome coronavirus 2, SARS-CoV-2, viruses, respiratory infections, zoonoses, healthcare personnel, risk factors, United States

## Abstract

To determine risk factors for coronavirus disease (COVID-19) among US healthcare personnel (HCP), we conducted a case–control analysis. We collected data about activities outside the workplace and COVID-19 patient care activities from HCP with positive severe acute respiratory syndrome coronavirus 2 (SARS-CoV-2) test results (cases) and from HCP with negative test results (controls) in healthcare facilities in 5 US states. We used conditional logistic regression to calculate adjusted matched odds ratios and 95% CIs for exposures. Among 345 cases and 622 controls, factors associated with risk were having close contact with persons with COVID-19 outside the workplace, having close contact with COVID-19 patients in the workplace, and assisting COVID-19 patients with activities of daily living. Protecting HCP from COVID-19 may require interventions that reduce their exposures outside the workplace and improve their ability to more safely assist COVID-19 patients with activities of daily living.

In the United States, ≈37 million cases of coronavirus disease (COVID-19) and >620,000 deaths had been reported as of June 30, 2021 (*1*). Given the critical role of healthcare personnel (HCP) in mitigating the COVID-19 pandemic, protecting them from severe acute respiratory syndrome coronavirus 2 (SARS-CoV-2) infection has been a focus of national and international response efforts. However, data on COVID-19 patient care activities that increase risk for SARS-CoV-2 infections among US HCP are limited. To describe factors associated with SARS-CoV-2 infection among US HCP, the Centers for Disease Control and Prevention (CDC) collaborated with Emerging Infections Program (EIP) site staff (*2*) to conduct a case–control analysis among HCP working in selected healthcare facilities. We assessed associations between SARS-CoV-2 infection in HCP and a variety of potential exposures: having close contact with persons with COVID-19 outside the workplace, having close contact with COVID-19 patients in the workplace, performing COVID-19 patient care activities including aerosol-generating procedures, and using recommended personal protective equipment (PPE) during those activities.

## Methods

### Healthcare Facilities and Personnel

Staff at EIP sites in 5 states (Colorado, Minnesota, New Mexico, New York, and Oregon) recruited a convenience sample of healthcare facilities and health systems to participate in the study. Eligible facilities included acute-care hospitals, nursing homes, or other healthcare facilities (e.g., outpatient clinics, urgent care clinics, or free-standing emergency departments). Healthcare personnel were defined as persons serving in healthcare settings with the potential for direct or indirect exposure to patients or infectious materials including body substances (e.g., blood, tissue, and specific body fluids); contaminated medical supplies, devices, and equipment; contaminated environmental surfaces; and contaminated air (*3*). This activity was reviewed by CDC and was conducted in compliance with applicable federal law and CDC policy (45 C.F.R. part 46.102(l)(2); 21 C.F.R. part 56; 42 U.S.C. §241(d); 5 U.S.C. §552a; 44 U.S.C. §3501 et seq.). CDC determined that the project was a nonresearch activity and no institutional review board review was required. EIP sites and participating facilities either deemed the project to be a nonresearch activity or obtained institutional review board approval.

### Case and Control Definitions and Enrollment

We defined cases as HCP working in participating healthcare facilities who had a positive SARS-CoV-2 PCR or antigen test (both of which are hereafter referred to as virus test) result from May 19, 2020, through December 6, 2020. To identify cases, EIP site staff obtained weekly lists of HCP with SARS-CoV-2 virus test results from participating healthcare facilities or state or local health departments. EIP site staff attempted to contact all HCP on the weekly lists and enroll all HCP meeting the case definition and agreeing to participate.

We defined controls as HCP who worked in participating healthcare facilities and had a negative SARS-CoV-2 virus test result during the same period used to define cases. To identify controls, EIP site staff randomly selected HCP with negative SARS-CoV-2 virus test results from the weekly lists of HCP test results provided by participating healthcare facilities or state or local health departments. HCP who had previously tested positive for SARS-CoV-2 were not eligible to be included as controls. EIP staff contacted randomly selected HCP and enrolled HCP who met the control definition and agreed to participate. 

We matched 2 controls to each case according to the healthcare facility in which the HCP worked and the week of collection of the SARS-CoV-2 virus test; we excluded unmatched cases and controls. To minimize recall bias, we did not enroll HCP if >60 days had elapsed since the specimen collection date for the SARS-CoV-2 virus test.

### Sample Size Calculation

The sample size estimate was based on the assumption that 50% of cases and controls would have had known close contact with COVID-19 patients in the workplace and that 15% of cases and 5% of controls would have participated (performed or assisted) in aerosol-generating procedures for COVID-19 patients. With a matched design and twice as many controls as cases, ≈200 cases and 400 controls would be required to detect an odds ratio of 2 as statistically significant with 80% power.

### Data Collection and Exposures of Interest

From May 28 through December 20, 2020, trained EIP staff conducted telephone interviews of HCP who agreed to participate; they used a standardized questionnaire to collect information about demographics, activities outside the workplace, detailed COVID-19 patient care activities including aerosol-generating procedures, and PPE use during those activities in the 14 days before specimen collection (asymptomatic HCP) or before COVID-19 symptom onset (symptomatic HCP). One case and 7 controls completed a self-administered questionnaire online.

Our primary exposures of interest were having close contact with persons with COVID-19 outside the workplace, having close contact with COVID-19 patients in the workplace, participating in aerosol-generating procedures for COVID-19 patients, performing selected COVID-19 patient care activities, and wearing recommended PPE during COVID-19 patient care activities. We grouped COVID-19 patient care activities into the following categories: assistance with activities of daily living (ADL; e.g., bathing, eating, toileting) or participating in restraining patients (hereafter, the phrase assisting with ADL also includes participating in restraint); clinical procedures (e.g., phlebotomy, intravenous line insertion, or a surgical procedure); nonprocedure clinical care (e.g., checking vital signs or performing a physical examination); environmental cleaning; respiratory care (e.g., nasal swabbing for SARS-CoV-2 testing, manipulating oxygen or ventilator tubing, or providing tracheostomy care); or administrative activities. Close contact was initially defined as being within ≈6 feet (≈2 m) of a person with SARS-CoV-2 infection for at least a few minutes or having unprotected direct contact with infectious secretions or excretions from the patient (*3*). However, to align with evolving guidance from CDC, we updated the definition of close contact twice during the project period (i.e., to specify a duration of 15 minutes and to include participation in aerosol-generating procedures regardless of duration) (*4*). Recommended PPE for COVID-19 patient care included gloves, gown, N95 respirator or powered air purifying respirator, and face shield or goggles. For this analysis, we considered the following activities to be aerosol-generating procedures according to CDC guidance: airway suctioning, sputum induction, cardiopulmonary resuscitation, endotracheal intubation or extubation, noninvasive positive pressure ventilation, bronchoscopy, and manual ventilation (*5*). We also included the following as aerosol-generating procedures because of the lack of data to definitively rule out potential aerosol generation: nebulizer administration, high-flow oxygen delivery, high-frequency oscillatory ventilation, chest physiotherapy, mini-bronchoalveolar lavage, and breaking the ventilation circuit in a patient receiving invasive mechanical ventilation (*6*).

Data were collected and managed by using REDCap (Research Electronic Data Capture) (*7*,*8*), a secure, web-based software platform designed to support data capture for research studies. It provides an intuitive interface for validated data capture, audit trails for tracking data manipulation and export procedures, automated export procedures for seamless data downloads to common statistical packages, and procedures for data integration and interoperability with external sources.

### Statistical Analyses

We summarized HCP characteristics by using frequencies for categorical variables and medians with interquartile ranges (IQRs) for continuous variables. To determine the variables to include in the multivariable conditional logistic regression models, we used direct acyclic graphs (*9*). We created 2 separate models to include appropriate HCP for the variables evaluated and calculated adjusted matched odds ratios (amORs) and 95% CIs for exposure variables.

Model 1 evaluated whether close contact with persons with COVID-19 outside the workplace or close contact with COVID-19 patients in the workplace was associated with SARS-CoV-2 infection in HCP. All cases and matched controls were included in the model, which was adjusted for HCP age, race and ethnicity, healthcare roles, and underlying medical conditions. Model 2 evaluated whether the following selected practices and activities in the workplace were associated with SARS-CoV-2 infection in HCP: participating in aerosol-generating procedures, performing different categories of COVID-19 patient care activities, or wearing recommended PPE during COVID-19 patient care activities. We included in model 2 only HCP who reported close contact with COVID-19 patients in the workplace. In this model, because of the small numbers of cases and controls when matching by week, we postmatched (*10*,*11*) by month of SARS-CoV-2 virus test specimen collection and controlled for HCP age, race and ethnicity, healthcare roles, underlying medical conditions, and close contact with persons with COVID-19 outside the workplace. To determine adequate model fit, we assessed Akaike information criteria and the presence of outliers, influential observations, and collinearity. We used SAS version 9.4 statistical software (https://www.sas.com) for the analyses.

## Results

The 25 participating healthcare facilities reported 33,644 HCP (3,416 cases and 30,228 controls) to EIP sites. Among 3,416 cases, 1,172 (34.3%) were interviewed, 1,070 (31.3%) did not respond to contact attempts or declined participation, and 1,174 (34.4%) were not interviewed because of other reasons (e.g., wrong telephone number or >60 days had elapsed since the specimen collection date for the SARS-CoV-2 virus test). Of the 1,172 cases who were interviewed, 345 (29.4%) were included in the case–control analysis on the basis of having >1 matched control and complete SARS-CoV-2 virus test data. Among 30,228 controls, 2,251 (7.4%) were selected to be contacted for an interview. Of these 2,251 HCP, 687 (30.5%) were interviewed, 1,174 (52.2%) did not respond to contact attempts or declined participation, and 390 (17.3%) were not interviewed because of other reasons. Among the 687 controls who were interviewed, 622 (90.5%) were included on the basis of having 1 matched case and complete SARS-CoV-2 virus test data. The median time from SARS-CoV-2 virus test specimen collection date to interview was 8 days (IQR 6–12) for cases and 16 days (IQR 10–26) for controls.

### Characteristics of Cases and Controls

Among the 967 HCP, 701 (72.5%) reported working in a hospital. Among the 345 cases, median age was 35 (IQR 28–47) years; 268 (77.7%) were female, 194 (56.2%) were White non-Hispanic, 96 (27.8%) were registered nurses, 127 (36.8%) reported close contact with persons with COVID-19 outside the workplace, and 113 (32.8%) reported close contact with COVID-19 patients in the workplace in the 14 days before illness onset or SARS-CoV-2 virus test specimen collection (Appendix Table 1). Approximately two thirds of cases and controls reported close contact with family members with COVID-19. Higher percentages of cases than controls identified themselves as being Hispanic or Latino, being <30 years of age, being administrative personnel, and having close contact with persons with COVID-19 outside the workplace (Appendix Tables 1–3). Obesity was more frequently reported by cases. The frequency of other underlying medical conditions was similar among cases and controls (Appendix Table 4).

### Close Contact with Persons with COVID-19 Outside the Workplace and COVID-19 Patients in the Workplace

According to the model 1 analysis, cases were significantly more likely than controls to report close contact with persons with COVID-19 outside the workplace (amOR 6.2, 95% CI 4.1–9.4; p<0.001) or close contact with COVID-19 patients in the workplace (amOR 1.6, 95% CI 1.1–2.3; p = 0.02), after controlling for HCP age, race and ethnicity, healthcare roles, and underlying medical conditions (Table 1).

**Table 1:** Multivariable conditional logistic regression model to identify characteristics, activities, and practices associated with SARS-CoV-2 infection among US healthcare personnel (model 1)*

Characteristic	No. (%)		amOR (95% CI)†	p value
	Cases, n = 345	Controls, n = 622		
Close contact with persons with COVID-19 outside the workplace‡				
No, unknown, or not reported§	218 (63.2)	560 (90)	Referent	
Yes	127 (36.8)	62 (10.0)	6.2 (4.1–9.4)	<0.001
Close contact with COVID-19 patients in the workplace‡¶				
No, unknown, or not reported	232 (67.2)	398 (68.3)	Referent	
Yes	113 (32.8)	197 (31.7)	1.6 (1.1–2.3)	0.02
Age#				
<30 y	107 (31.0)	143 (23.0)	Referent	
≥30 y	238 (69.0)	473 (76.1)	0.7 (0.5–1.0)	0.04
Any underlying medical condition(s)**				
No	112 (32.5)	222 (35.7)	Referent	
Yes	233 (67.5)	400 (64.3)	1.3 (0.9–1.8)	0.12
Race and ethnicity††				
White, non-Hispanic	194 (56.2)	406 (65.3)	Referent	
Hispanic or Latino	86 (24.9)	106 (17.0)	1.7 (1.1–2.6)	0.02
Black, non-Hispanic	25 (7.2)	28 (4.5)	1.7 (0.9–3.2)	0.12
Asian, non-Hispanic	17 (4.9)	29 (4.7)	1.2 (0.6–2.5)	0.56
Other or multiple races, non-Hispanic, or race or ethnicity not reported	23 (6.8)	53 (8.5)	0.9 (0.5–1.8)	0.81
Healthcare role				
Registered nurse	96 (27.8)	201 (32.3)	Referent	
Administrative personnel	47 (13.6)	50 (8.0)	1.8 (1.1–3.2)	0.04
Physician	20 (5.8)	63 (10.1)	0.9 (0.5–1.7)	0.73
Nursing assistant or patient care technician	24 (7.0)	36 (5.8)	1.1 (0.6–2.2)	0.78
Medical assistant	16 (4.6)	23 (3.7)	1.1 (0.5–2.5)	0.88
Other role anticipated to have substantial patient contact‡‡	58 (16.8)	107 (17.2)	0.9 (0.6–1.5)	0.83
Other role anticipated to have moderate patient contact§§	51 (14.8)	77 (12.4)	1.1 (0.7–1.9)	0.70
Other role anticipated to have minimal patient contact¶¶	24 (7.0)	36 (5.8)	1.4 (0.7–2.7)	0.32
Other role with undefined level of patient contact	9 (2.6)	29 (4.7)	0.6 (0.2–1.4)	0.25

*amOR, adjusted matched odds ratio; COVID-19, coronavirus disease; IQR, interquartile range; SARS-CoV-2, severe acute respiratory syndrome coronavirus 2. †Model included 967 healthcare personnel: 345 cases and 622 controls. Among these, there were 71 pairs of 1 case and 1 control, 271 clusters of 1 case and 2 controls, and 3 clusters of 1 case and 3 controls. ‡In the 14 d before illness onset or SARS-CoV-2 virus test specimen collection date. §15 cases and 14 controls reported that they did not know if they had close contact with persons with COVID-19 outside the workplace; data were missing for 1 control. ¶15 cases and 27 controls reported that they did not know if they had close contact with COVID-19 patients in the workplace; data were missing for 1 control. #Age was not reported for 6 controls; these healthcare personnel were grouped as <30 y. **Asthma, rhinitis, chronic obstructive pulmonary disease or other chronic lung diseases, hypertension or heart conditions, diabetes mellitus, chronic kidney disease or hemodialysis, autoimmune or rheumatologic disease, active cancer, solid organ or hematopoietic stem cell transplant, other immunosuppressing conditions, chronic liver disease, pregnancy, current or recent smoking (i.e., within a year of SARS-CoV-2 virus test specimen collection date), and obesity or severe obesity with body mass index >30. ††Race was not reported for 16 cases and 27 controls; ethnicity was missing for 14 cases and 24 controls. ‡‡Dental healthcare provider, emergency medical services personnel, licensed practical nurse, nurse practitioner, occupational therapist, other nurse, physician assistant, physical therapist or assistant, phlebotomist, respiratory therapist, radiology technician, speech-language pathologist, and surgical, medical, or emergency technician. §§Nonphysician behavioral health provider, chaplain, care coordinator, dietician, environmental services personnel, food services personnel, patient transport personnel, research personnel, social worker, or student. ¶¶Facilities maintenance personnel, medical equipment technician, laboratory personnel, or pharmacist. Detailed healthcare roles and area of the facility in which HCP worked are available in Appendix Tables 2 and 3 (https://wwwnc.cdc.gov/EID/article/28/1/21-1803-App1.pdf).

### Characteristics of HCP Reporting Close Contact with Patients with COVID-19 in the Workplace

Among the 310 HCP who reported close contact with COVID-19 patients in the workplace, cases and controls reported performing similar patient care activities, except a higher percentage of cases (69.9%) than controls (54.3%) reported assisting COVID-19 patients with their ADL (Appendix Table 5). Of the 87 (28.4%) HCP who participated in aerosol-generating procedures, the proportion of cases and controls who reported wearing all recommended PPE all the time varied by the type of aerosol-generating procedure. Of note, the percentages of cases who reported wearing all recommended PPE all the time during common aerosol-generating procedures such as noninvasive positive pressure ventilation, manual ventilation, nebulization administration, or high-flow oxygen delivery were lower than the percentages of controls who reported the same (Appendix Table 5).

### COVID-19 Patient Care Activities and PPE Use

According to the model 2 analysis, 274 (88.4%) of 310 HCP who reported close contact with COVID-19 patients in the workplace were postmatched. After controlling for HCP age, race and ethnicity, healthcare roles, underlying medical conditions, and close contact with persons with COVID-19 outside the workplace, cases were significantly more likely than controls to report assisting COVID-19 patients with their ADL (amOR 4.7, 95% CI 1.7–12.7; p = 0.003); however, no differences in aerosol-generating procedure participation (amOR 0.9, 95% CI 0.4–2.0; p = 0.84) or wearing recommended PPE all the time during COVID-19 patient care activities (amOR 0.9, 95% CI 0.5–2.0; p = 0.88) were identified among cases and controls (Table 2).

**Table 2:** Multivariable conditional logistic regression model to identify activities and practices associated with SARS-CoV-2 infection among US healthcare personnel who reported caring for COVID-19 patients in the workplace (model 2)*

Characteristic	No. (%)		amOR (95% CI)†	p value
	Cases, n = 105	Controls, n = 169		
Participated in AGP				
No, unknown, or missing	72 (68.6)	123 (72.8)	Referent	
Yes	33 (31.4)	47 (27.8)	0.9 (0.4–2.0)	0.84
Wore recommended personal protective equipment all the time during non-AGP COVID-19 patient care				
No, unknown, or missing	67 (63.8)	110 (65.1)	Referent	
Yes	38 (36.2)	59 (34.9)	0.9 (0.5–2.0)	0.88
Assisted COVID-19 patients with activities of daily living				
No	31 (29.5)	79 (46.8)	Referent	
Yes	74 (70.5)	90 (53.2)	4.7 (1.7–12.7)	0.003
Provided nonprocedure clinical care to COVID-19 patients				
No	19 (18.1)	35 (20.7)	Referent	
Yes	86 (81.9)	134 (79.3)	0.9 (0.3–2.6)	0.88
Performed procedures on COVID-19 patients				
No	56 (53.3)	84 (49.7)	Referent	
Yes	49 (46.7)	85 (50.3)	0.6 (0.3–1.4)	0.25
Performed environmental cleaning activities in COVID-19 patient care area				
No	53 (50.5)	100 (59.2)	Referent	
Yes	52 (49.5)	69 (40.8)	1.2 (0.5–2.8)	0.69
Provided respiratory care to COVID-19 patients				
No	51 (48.6)	88 (52.1)	Referent	
Yes	54 (51.4)	81 (47.9)	0.8 (0.3–2.0)	0.70
Performed administrative activities with COVID-19 patients				
No	94 (89.5)	149 (88.2)	Referent	
Yes	11 (10.5)	20 (11.8)	0.9 (0.3–3.2)	0.90
Close contact with persons with COVID-19 outside the workplace				
No, unknown, or missing	86 (81.9)	160 (94.7)	Referent	
Yes	19 (18.1)	9 (5.3)	4.9 (1.7–13.9)	0.003
Healthcare role				
Registered nurse	39 (37.1)	69 (40.8)	Referent	
Administrative personnel	3 (2.9)	6 (3.6)	2.6 (0.3–24.8)	0.40
Nursing assistant or patient care technician	10 (9.5)	16 (9.5)	0.4 (0.1–1.6)	0.22
Physician	4 (3.8)	23 (13.6)	0.6 (0.1–3.7)	0.60
Medical assistant	3 (2.9)	4 (2.4)	1.7 (0.2–12.5)	0.60
Other role anticipated to have substantial patient contact	36 (34.3)	35 (20.7)	2.6 (1.1–6.5)	0.04
Other role anticipated to have moderate patient contact	6 (5.7)	10 (5.9)	1.3 (0.2–7.9)	0.75
Other role anticipated to have minimal patient contact	2 (1.9)	2 (1.2)	4.1 (0.2–78.4)	0.35
Other role with undefined level of patient contact	2 (1.9)	4 (2.4)	0.7 (0.1–6.6)	0.73
Race and ethnicity				
White, non-Hispanic	63 (60.0)	102 (60.4)	Referent	
Hispanic or Latino	31 (29.5)	34 (20.1)	2.1 (0.9–4.8)	0.07
Asian, non-Hispanic	4 (3.8)	8 (4.7)	0.8 (0.2–3.8)	0.83
Black, non-Hispanic	3 (2.9)	6 (3.6)	0.7 (0.1–4.4)	0.73
Other or multiple races, non-Hispanic or race or ethnicity not reported	4 (3.8)	19 (11.2)	0.29 (0.1–1.2)	0.09
Any underlying condition(s)				
No	26 (24.8)	62 (36.7)	Referent	
Yes	79 (75.2)	107 (63.3)	2.5 (1.2–5.1)	0.013
Age, y‡				
<30	67 (63.8)	127 (75.2)	Referent	
>30	38 (36.2)	42 (24.8)	0.5 (0.2–0.9)	0.036

*AGP, aerosol-generating procedures; amOR, adjusted matched odds ratio; COVID-19, coronavirus disease. †Model included 274 HCP (105 cases and 169 controls). HCP were postmatched into 47 clusters, each cluster with >1 case and >1 control, and the largest cluster with 10 cases and 20 controls. ‡Age was not reported for 3 controls; these HCP were grouped as <30 y.

## Discussion

Our analysis included 967 US HCP from 21 healthcare facilities in 5 US states and used data from interviews conducted before widespread availability of COVID-19 vaccines. After controlling for demographic characteristics, healthcare roles, and underlying medical conditions, we found that compared with matched controls, odds for cases were 6.2-fold higher for reporting close contact with persons with COVID-19 outside the workplace; 1.6-fold higher for reporting close contact with COVID-19 patients in the workplace; and, among HCP who reported close contact with COVID-19 patients in the workplace, 4.7-fold higher for assisting COVID-19 patients with their ADL.

The greater odds of cases reporting close contact with persons with COVID-19 outside the workplace is consistent with findings of multiple studies, such as studies by Lentz et al., which included >1,600 HCP from 67 countries, and by Jacob et al., which included >24,000 HCP from 4 large healthcare systems in 3 US states (*12*–*20*). Our analysis also showed that most cases reported close contact with family members with COVID-19. This finding underscores the value of interventions aimed at mitigating community transmission of SARS-CoV-2, particularly among racial and ethnic minority groups that have been disproportionately affected by COVID-19 (*21*,*22*).

Some studies have not identified an association between SARS-CoV-2 infection in HCP and close contact with COVID-19 patients in the workplace (*13*–*18*). Our analysis, however, showed that assisting COVID-19 patients with ADL was independently associated with being an HCP case. HCP might be less likely to adhere to infection prevention measures during patient care activities that are not perceived to be high risk compared with activities such as aerosol-generating procedures. In addition, during periods of PPE shortages, healthcare facilities may have reserved selected PPE, such as N95 respirators, for HCP in certain roles or those participating in aerosol-generating procedures, restricting the availability of some protective equipment for use when performing other patient care tasks perceived to be less risky. Continued reinforcement of recommended infection prevention measures in healthcare settings, especially during activities that require prolonged close contact with COVID-19 patients, is needed. Future studies may better describe COVID-19 patient care activities that pose the greatest risk to HCP and are most amenable to interventions.

In a scientific brief dated May 7, 2020, CDC described the 3 modes of SARS-CoV-2 transmission: “1) inhalation of very fine respiratory droplets and aerosol particles, 2) deposition of respiratory droplets and particles on exposed mucous membranes in the mouth, nose, or eye by direct splashes and sprays, and 3) touching mucous membranes with hands that have been soiled either directly by virus-containing respiratory fluids or indirectly by touching surfaces with virus on them” (*23*). Based on experience with SARS-CoV-1, there have been concerns that risk for infection may be higher for HCP who participate in aerosol-generating procedures than those who do not because of the proximity and time spent with patients and the large quantity of aerosol particles generated during such procedures (*24*). We did not detect a difference in reported aerosol-generating procedure participation between cases and controls, which is consistent with results reported by Lentz et al. (*12*). This lack of association may be explained by HCP use of effective infection prevention measures (*25*–*28*) implemented since the start of the pandemic, including use of recommended PPE. It should be noted that our analysis included a broader definition of aerosol-generating procedure than CDC and World Health Organization guidance (*27*,*28*). Recent studies showed that some aerosol-generating procedures included in public health guidance, such as intubation and extubation, generated negligible amounts of aerosols if performed on asymptomatic patients in controlled settings (*29*,*30*).

Assessing the effect of PPE use on SARS-CoV-2 transmission is challenging, especially when reported use is based on HCP recall rather than observation. HCP may have reported having used recommended PPE all the time even when they had not, knowing that this was the socially desirable response. Such reporting bias might explain why we did not observe a protective effect of wearing recommended PPE all the time for HCP engaged in COVID-19 patient care activities, a finding that has been reported by others (*31*). Other potential explanations for the lack of association between PPE use and COVID-19 case status among HCP include the small numbers of cases and controls in our analysis, limiting our ability to detect statistically significant differences; HCP participation in multiple patient care activities that may have placed them at risk, including aerosol-generating procedures; and the inability to assess whether PPE was used correctly. Other prevention measures, such as use of source control for patients and performing activities in airborne infection isolation rooms, may have masked the effect of PPE use on transmission of SARS-CoV-2 to HCP (*27*).

The first limitation of our study is that testing practices and test types may have varied among participating facilities. The exact accuracy of the tests used was unknown and was not accounted for in our analysis. A small number of cases and controls could have been misclassified on the basis of false-positive or false-negative results. A second limitation is that although the minimum target sample size was achieved, the percentages of HCP who reported close contact with COVID-19 patients in the workplace were lower than those used for the sample size calculation, limiting our ability to detect significant differences in workplace exposures between cases and controls. Third, we included a convenience sample of healthcare facilities at 5 EIP sites, and most participating facilities were acute-care hospitals. In addition, although controls were randomly selected to be contacted for interview, the cases and controls who responded to contact attempts and agreed to participate might not be representative of all US HCP and, therefore, results may not be generalizable to all US HCP. Fourth, differential exposure misclassification may have resulted from the time that elapsed between HCP SARS-CoV-2 virus test specimen collection and the interview. The time from SARS-CoV-2 virus test specimen collection to interview was longer for controls than for cases. In addition, cases may have been more likely than controls to remember close contact with persons with COVID-19, resulting in recall bias. Fifth, our analysis included practices and activities conducted by HCP before COVID-19 vaccines were available and before detection of the Delta variant. Because of evolving infection prevention and control guidance, testing practices, vaccine availability, and SARS-CoV-2 variant emergence, the risk factors identified in this analysis should be interpreted in the context of current guidance and knowledge.

In conclusion, according to data gathered from HCP interviews conducted before widespread availability of COVID-19 vaccines, HCP cases reported more frequent close contact with persons with COVID-19 outside the workplace and COVID-19 patients in the workplace than did HCP controls. These findings suggest that in addition to vaccination and healthcare infection prevention and control measures, protecting HCP requires interventions that reduce HCP exposures to SARS-CoV-2 in their communities. Among HCP who provided care for COVID-19 patients, cases reported more frequently assisting COVID-19 patients with ADL than did controls. Protecting HCP may require interventions that reduce COVID-19 exposures outside the workplace and improve HCP’s ability to assist COVID-19 patients with ADL more safely. Infection control training programs and measures specifically focused on protecting HCP may be particularly useful for reducing SARS-CoV-2 transmission in healthcare facilities.

Appendix Supplemental results from analysis of risk factors for SARS-CoV-2 infection among US healthcare personnel, May–December 2020.
