# Supplementary material for: Risk Factors for SARS-CoV-2 Infection Among US Healthcare Personnel, May–December 2020
Source: Emerg Infect Dis. 2022 Jan;28(1):95–103. doi: 10.3201/eid2801.211803 (PMC8714235; doi:10.3201/eid2801.211803)
Supplement: Appendix — Supplemental results from analysis of risk factors for SARS-CoV-2 infection among US healthcare personnel, May–December 2020. [file 21-1803-Techapp-s1.pdf]

Article DOI: https://doi.org/10.3201/eid2801.211803

Risk Factors for SARS-CoV-2 Infection Among US Healthcare Personnel, May–December 2020

Appendix

Appendix Table 1. Characteristics of healthcare personnel with (cases) and without (controls) SARS-CoV-2 infection, 5 US Emerging Infections Program sites, May–December 2020*

Characteristic	Cases (n = 345)	Controls (n = 622)
Site, no. (%)		
Colorado	89 (25.8)	165 (26.5)
Minnesota	51 (14.8)	86 (13.8)
New Mexico	57 (16.5)	101 (16.2)
New York	52 (15.1)	98 (15.8)
Oregon	96 (27.8)	172 (27.7)
Facility type, no. (%)		
Hospital	269 (78.0)	432 (69.5)
Nursing home	17 (4.9)	37 (5.9)
Other facility type†	59 (17.1)	151 (24.3)
Not reported	–	2 (0.3)
Days from SARS-CoV-2 antigen test specimen collection date to interview, median (IQR)	8 (6–12)	16 (10–26)
Days from COVID-19 symptom onset to SARS-CoV-2 antigen test specimen collection, median (IQR)‡	2 (1–4)	2 (1–4)
Age, y, median (IQR)	35 (28–47)	37 (30–47)
Age group, y, no. (%)		
<30	107 (31.0)	143 (23.0)
≥30	238 (69.0)	473 (76.1)
Not reported	–	6 (0.9)
Sex, no. (%)		
F	268 (77.7)	473 (76.0)
M	77 (22.3)	142 (22.8)
Other or not reported	–	7 (1.1)
Race and ethnicity, no. (%)		
White, non-Hispanic	194 (56.2)	406 (65.3)
Hispanic or Latino, any race or races	86 (24.9)	106 (17.0)
Black, non-Hispanic	25 (7.2)	28 (4.5)
Asian, non-Hispanic	17 (4.9)	29 (4.7)
Other or multiple races, non-Hispanic or race or ethnicity not reported	23 (6.8)	53 (8.5)
Healthcare role, no. (%)		
Registered nurse	96 (27.8)	201 (32.3)
Administrative personnel	47 (13.6)	50 (8.0)
Physician	20 (5.8)	63 (10.1)
Nursing assistant or patient care technician	24 (7.0)	36 (5.8)
Medical assistant	16 (4.6)	23 (3.7)
Other role anticipated to have substantial patient contact§	58 (16.8)	107 (17.2)
Other role anticipated to have moderate patient contact¶	51 (14.8)	77 (12.4)
Other role anticipated to have minimal patient contact#	24 (7.0)	36 (5.8)
Other role with undefined level of patient contact	9 (2.6)	29 (4.7)
≥1 underlying medical condition, no. (%)**	233 (67.5)	400 (64.3)
≥1 symptom in the 14 d before or on SARS-CoV-2 antigen test specimen collection, no. (%)††	331 (95.9)	390 (62.7)
Close contact with patients with COVID-19 in the workplace in the 14 d before illness onset or SARS-CoV-2 antigen test specimen collection date, no. (%)		
Yes	113 (32.8)	197 (31.7)
No	217 (62.9)	397 (63.8)
Unknown or not reported	15 (4.3)	28 (4.5)
Close contact with persons with COVID-19 outside the workplace in the 14 d before illness onset or SARS-CoV-2 antigen test specimen collection date, no. (%)	127 (36.8)	62 (10.0)

Characteristic	Cases (n = 345)	Controls (n = 622)
Yes—family member	83 (24.1)	39 (6.3)
Spouse or partner	40 (11.6)	16 (2.6)
Child	9 (2.6)	10 (1.6)
Parent	16 (4.6)	4 (0.6)
Other family member	23 (6.7)	11 (1.8)
Yes—someone else (not a family member) with COVID-19	47 (13.6)	23 (3.7)
No	203 (58.8)	545 (87.6)
Unknown or not reported	15 (4.3)	15 (2.5)
Travel, no. (%)		
Yes	78 (22.6)	148 (23.8)
International	5 (1.4)	1 (0.2)
Domestic	73 (21.2)	147 (23.6)
No	265 (76.8)	472 (75.9)
Unknown or not reported	2 (0.6)	2 (0.3)
Attended a gathering that included persons other than household members, no. (%)		
Yes	90 (26.1)	158 (25.4)
No	255 (73.9)	461 (74.1)
Unknown or not reported	0 (0)	3 (0.5)
Used public transportation, no. (%)		
Yes	31 (9.0)	62 (10.0)
No	314 (91.0)	559 (89.9)
Unknown or not reported	0 (0)	1 (0.2)
Used ride share service, no. (%)		
Yes	29 (8.4)	40 (6.4)
No	313 (90.7)	580 (93.2)
Unknown or not reported	3 (0.9)	2 (0.4)
Practiced physical distancing with co-workers in the workplace, no. (%)		
Always	83 (24.1)	89 (14.3)
Most of the time	136 (39.4)	232 (37.3)
Some of the time	63 (18.3)	157 (25.2)
Never	62 (18.0)	140 (22.5)
Not reported	1 (0.3)	4 (0.6)
Practiced universal mask wearing in the workplace, no. (%)		
Always	290 (84.1)	517 (83.1)
Most of the time	44 (12.8)	86 (13.8)
Some of the time	3 (0.9)	9 (1.4)
Never	7 (2.0)	6 (1.0)
Not reported	1 (0.3)	4 (0.6)

*COVID-19, coronavirus disease; IQR, interquartile range; SARS-CoV-2, severe acute respiratory syndrome coronavirus 2.

†Includes outpatient clinics, urgent care clinics, free-standing emergency rooms or departments, and mental health facilities.

‡Of 345 cases, 305 were symptomatic with onset date before positive test specimen collection date and included in the calculations; of 622 controls, 382 were symptomatic with onset date before negative test specimen collection date and included in the calculations. Symptoms include fever, chills, dry or productive cough, fatigue or malaise, sore throat, runny nose, shortness of breath, muscle aches, headache, chest pain or tightness, nausea or vomiting, diarrhea, abdominal pain, altered sense of smell or taste, congestion, loss of appetite.

§Includes dental healthcare provider, emergency medical services personnel, licensed practical nurse, nurse practitioner, occupational therapist, other nurse, physician assistant, physical therapist or assistant, phlebotomist, respiratory therapist, radiology technician, speech-language pathologist, and surgical, medical, or emergency technician.

¶Includes nonphysician behavioral health provider, chaplain, care coordinator, dietician, environmental services personnel, food services personnel, patient transport personnel, research personnel, social worker, or student.

#Includes facilities maintenance personnel, medical equipment technician, laboratory personnel, or pharmacist. Detailed healthcare roles and area of the facility in which healthcare personnel worked are available in Appendix Tables 2 and 3 (https://wwwnc.cdc.gov/EID/article/28/1/21-1803-App1.pdf).

**Includes asthma, rhinitis, chronic obstructive pulmonary disease or other chronic lung diseases, hypertension or heart conditions, diabetes mellitus, chronic kidney disease or hemodialysis, autoimmune or rheumatologic disease, active cancer, solid organ or hematopoietic stem cell transplant, other immunosuppressing conditions, chronic liver disease, pregnancy, current or recent smoking (i.e., within a year of SARS-CoV-2 antigen test specimen collection), and obesity or severe obesity with body mass index $\geq$30. Detailed underlying medical conditions are available in Appendix Table 4.

††Includes fever, chills, dry or productive cough, fatigue or malaise, sore throat, runny nose, shortness of breath, muscle aches, headache, chest pain or tightness, nausea or vomiting, diarrhea, abdominal pain, altered sense of smell or taste, congestion, loss of appetite.

Appendix Table 2. Roles of healthcare personnel cases and controls, May–December 2020

Healthcare role, no. (%)	Cases (N=345)	Controls (N=622)
Registered nurse	96 (27.8)	201 (32.3)
Administrative personnel	47 (13.6)	50 (8.0)
Physician	20 (5.8)	63 (10.1)
Nursing assistant or patient care technician	24 (7.0)	36 (5.8)
Medical assistant	16 (4.6)	23 (3.7)
Food services staff	17 (4.9)	18 (2.9)
Pharmacy personnel	5 (1.4)	20 (3.2)
Surgical or medical technician	9 (2.6)	12 (1.9)
Mental health personnel	4 (1.2)	16 (2.6)
Environmental services staff	13 (3.8)	7 (1.1)
Nurse practitioner	4 (1.2)	16 (2.6)
Radiology personnel	10 (2.9)	10 (1.6)
Physical therapy personnel	7 (2.0)	12 (1.9)
Laboratory personnel	8 (2.3)	9 (1.4)
Physician assistant	3 (0.9)	13 (2.1)
Social worker	3 (0.9)	12 (1.9)
Phlebotomist	3 (0.9)	11 (1.8)
Research personnel	6 (1.7)	8 (1.3)
Emergency medical service personnel	5 (1.4)	7 (1.1)
Facility personnel	6 (1.7)	5 (0.8)
Licensed practical nurse	3 (0.9)	8 (1.3)
Occupational therapy personnel	7 (2.0)	4 (0.6)
Respiratory therapist	4 (1.2)	7 (1.1)
Transport personnel	4 (1.2)	4 (0.6)
Medical equipment technician	5 (1.4)	2 (0.3)
Care manager or coordinator	3 (0.9)	3 (0.5)
Speech language pathologist	1 (0.3)	5 (0.8)
Nutritionist or nutrition assistant	1 (0.3)	4 (0.6)
Student	0 (0)	4 (0.6)
Nurse, other	2 (0.6)	1 (0.2)
Chaplain	0 (0)	1 (0.2)
Dental personnel	0 (0)	1 (0.2)
Other	9 (2.6)	28 (4.5)
Not reported	0 (0)	1 (0.2)

Appendix Table 3. Area of the healthcare facility in which healthcare personnel (HCP) worked, May–December 2020

Area of the facility in which the HCP worked, no. (%)	Case-HCP (N=345)	Control-HCP (N=622)
Inpatient ward	128 (37.1)	252 (40.5)
Outpatient clinic	70 (20.3)	170 (27.3)
Intensive care unit	60 (17.4)	118 (19.0)
Emergency department	64 (18.6)	105 (16.9)
Administrative offices	45 (13.0)	72 (11.6)
Reception area	30 (8.7)	51 (8.2)
Operating room	24 (7.0)	54 (8.7)
Nursing home ward	17 (4.9)	35 (5.6)
Radiology department	20 (5.8)	31 (5.0)
Laboratory	18 (5.2)	28 (4.5)
Dining room or cafeteria	17 (4.9)	20 (3.2)
Kitchen	15 (4.3)	14 (2.3)
Pharmacy	3 (0.9)	22 (3.5)
Home health or private residence	2 (0.6)	7 (1.1)
Endoscopy area	4 (1.2)	4 (0.6)
Other area	53 (15.4)	77 (12.4)

Appendix Table 4. Underlying medical condition of healthcare personnel, May–December 2020

Underlying medical conditions, no. (%)	Case-HCP (N=345)	Control-HCP (N=622)
Obesity or severe obesity	109 (31.6)	141 (22.7)
Hypertension	34 (9.9)	64 (10.3)
Asthma	53 (15.4)	98 (15.8)
Diabetes mellitus	13 (3.8)	20 (3.2)
Current or recent smoker	17 (4.9)	39 (6.3)
Autoimmune or rheumatologic disease	26 (7.5)	45 (7.2)
Heart condition	8 (2.3)	23 (3.7)
Pregnancy	6 (1.7)	14 (2.3)
Cancer	2 (0.6)	4 (0.6)
Chronic obstructive pulmonary disease	1 (0.3)	3 (0.5)
Chronic kidney disease	--	4 (0.6)
Other immunosuppression*	10 (2.9)	14 (2.3)

*Includes history of solid organ or hematopoietic stem cell transplantation, asplenia, and receipt of immunosuppressing medications.

Appendix Table 5. Characteristics of 310 US healthcare personnel who had close contact with COVID-19 patients in the workplace, May–December 2020*

Characteristic	Cases (n = 113)	Controls (n = 197)
Activities performed by healthcare personnel in the workplace with COVID-19 patients, no. (%)		
Nonprocedure clinical care†	91 (80.5)	151 (76.6)
Assistance with activities of daily living‡	79 (69.9)	107 (54.3)
Procedures§	52 (46.0)	100 (50.8)
Respiratory care activities¶	58 (51.3)	92 (46.7)
Environmental activities#	56 (49.6)	82 (41.6)
Administrative activities**	12 (10.6)	24 (12.2)
Personal protective equipment used by healthcare personnel during care of COVID-19 patients, no. (%)		
Gloves		
All of the time	102 (90.3)	161 (81.7)
Most of the time	4 (3.5)	12 (6.1)
Some of the time	0	10 (5.1)
Never	6 (5.3)	13 (6.6)
Not reported	1 (0.9)	1 (0.5)
Gown		
All of the time	79 (69.9)	107 (54.3)
Most of the time	6 (5.3)	18 (9.1)
Some of the time	10 (8.8)	20 (10.2)
Never	16 (14.2)	51 (25.9)
Not reported	2 (1.8)	1 (0.5)
N95 respirator		
All of the time	48 (42.5)	82 (41.6)
Most of the time	5 (4.4)	13 (6.6)
Some of the time	12 (10.6)	22 (11.2)
Never	47 (41.6)	76 (38.6)
Not reported	1 (0.9)	4 (2.0)
Powered air purifying respirator		
All of the time	2 (1.8)	5 (2.5)
Most of the time	2 (1.8)	3 (1.5)
Some of the time	0	7 (3.6)
Never	107 (94.7)	175 (88.8)
Not reported	2 (1.8)	7 (3.6)
Facemask		
All of the time	77 (68.1)	120 (60.9)
Most of the time	2 (1.8)	11 (5.6)
Some of the time	5 (4.4)	17 (8.6)
Never	29 (25.7)	46 (23.4)
Not reported	0	3 (1.5)
Goggles or face shield		
All of the time	80 (70.8)	128 (65.0)
Most of the time	6 (5.3)	15 (7.6)
Some of the time	5 (4.4)	21 (10.7)
Never	21 (18.6)	31 (15.7)
Not reported	1 (0.9)	2 (1.0)
All recommended personal protective equipment††		
All of the time	39 (34.5)	62 (31.5)
Not all of the time or not reported	74 (65.5)	135 (68.5)

Characteristic	Cases (n = 113)	Controls (n = 197)
Participation in aerosol-generating procedures, no. (%)		
Yes	35 (31.0)	53 (26.9)
High-flow oxygen delivery	22 (19.5)	32 (16.2)
Airway suctioning	19 (16.8)	30 (15.2)
Intubation or cardiopulmonary resuscitation	14 (12.4)	31 (15.7)
Noninvasive positive pressure ventilation	15 (13.3)	23 (11.7)
Nebulizer treatment	16 (14.2)	19 (9.6)
Manual (bag) ventilation	11 (9.7)	16 (8.1)
Breaking ventilation circuit	10 (8.8)	15 (7.6)
Chest physiotherapy	5 (4.4)	1 (0.5)
Mini bronchoalveolar lavage	2 (1.8)	3 (1.5)
Sputum induction	3 (2.7)	1 (0.5)
High frequency oscillatory ventilation	2 (1.8)	1 (0.5)
Bronchoscopy	2 (1.8)	1 (0.5)
No or unknown‡‡	78 (69.0)	145 (73.6)
Participation in aerosol-generating procedures, wearing all recommended personal protective equipment all the time, no. (%)	21 (18.4)	36 (18.6)
High-flow oxygen delivery§§	14/22 (63.6)	23/32 (71.9)
Airway suctioning§§	14/19 (73.7)	22/30 (73.3)
Intubation, extubation, or cardiopulmonary resuscitation§§	10/14 (71.4)	21/31 (67.7)
Noninvasive positive pressure ventilation§§	7/15 (46.7)	16/23 (69.6)
Nebulizer treatment§§	7/16 (43.8)	15/19 (78.9)
Manual (bag) ventilation§§	4/11 (36.4)	12/16 (75.0)
Breaking ventilation circuit§§	8/10 (80.0)	11/15 (73.3)
Chest physiotherapy§§	4/5 (80.0)	0/1 (0)
Mini bronchoalveolar lavage§§	2/2 (100)	3/3 (100)
Sputum induction§§	2/3 (66.7)	0/1 (0)
High frequency oscillatory ventilation§§	2/2 (100)	1/1 (100)
Bronchoscopy§§	2/2 (100)	1/1 (100)

*COVID-19, coronavirus disease.
†Includes physical examination, performing electrocardiograms or radiology tests, physical therapy, and providing medications.
‡Assistance with activities of daily living includes bathing, toileting, assisting with mobility, feeding, and participating in restraining patients.
§Include surgery, phlebotomy, suturing, wound care, endoscopy, and inserting or manipulating tubes, lines, or drains.
¶Respiratory care activities were considered separately from aerosol-generating procedures and include collecting respiratory specimens, manipulation of oxygen face mask, ventilator or tubing, tracheostomy care, and assisting with breathing exercises or speech and swallowing.
#Include cleaning patient rooms, changing linens, and performing room or equipment maintenance.
**Include interviews or verbal assessments, scheduling, registration, teaching, and delivering personal care items or food to the patient.
††Defined as reporting always wearing an N95 respirator or always wearing a PAPR, always wearing goggles or a face shield, always wearing a gown, and always wearing gloves when caring for patients with COVID-19.
‡‡Four controls reported unknown participation in aerosol-generating procedures.
§§ No. HCP who participated and wore all recommended PPE during the procedure/no. HCP who participated in the procedure.

.
